# Use of Quantile Treatment Effects Analysis to Describe Antidepressant Response in Randomized Clinical Trials Submitted to the US Food and Drug Administration: A Secondary Analysis of Pooled Trial Data

**DOI:** 10.1001/jamanetworkopen.2023.17714

**Published:** 2023-06-09

**Authors:** William U. Meyerson, Carl F. Pieper, Rick H. Hoyle

**Affiliations:** 1 Department of Psychiatry & Behavioral Sciences, Duke University Medical Center, Durham, North Carolina; 2 Department of Molecular Biophysics & Biochemistry, Yale University, New Haven, Connecticut; 3 Department of Biostatistics and Bioinformatics, Duke University Medical Center, Durham, North Carolina; 4 Center on Aging and Human Development, Duke University Medical Center, Durham, North Carolina; 5 Department of Psychology & Neuroscience, Duke University, Durham, North Carolina

## Abstract

**Importance:**

Major depressive disorder (MDD) is a leading cause of global distress and disability. Earlier studies have indicated that antidepressant therapy confers a modest reduction in depressive symptoms on average, but the distribution of this reduction requires more research.

**Objective:**

To estimate the distribution of antidepressant response by depression severity.

**Design, Setting, and Participants:**

In this secondary analysis of pooled trial data, quantile treatment effect (QTE) analysis was conducted from the US Food and Drug Administration (FDA) database of antidepressant monotherapy for patients with MDD, encompassing 232 positive and negative trials submitted to the FDA between 1979 and 2016. Analysis was restricted to participants with severe MDD (17-item Hamilton Rating Scale for Depression [HAMD-17] score ≥20). Data analysis was conducted from August 16, 2022, to April 16, 2023.

**Intervention:**

Antidepressant monotherapy compared with placebo.

**Main Outcomes and Measures:**

The distribution of percentage depression response was compared between the pooled treatment arm and pooled placebo arm. Percentage depression response was defined as 1 minus the ratio of final depression severity to baseline depression severity, expressed as a percentage. Depression severity was reported in HAMD-17-equivalent units.

**Results:**

A total of 57 313 participants with severe depression were included in the analysis. There was no significant imbalance in baseline depression severity between the pooled treatment arm and pooled placebo arm, with a mean HAMD-17 difference of 0.037 points (*P* = .11 by Wilcoxon rank sum test). An interaction term test for rank similarity did not reject the rank similarity governing percentage depression response (*P* > .99). The entire distribution of depression response was more favorable in the pooled treatment arm than in the pooled placebo arm. The maximum separation between treatment and placebo occurred at the 55th quantile and corresponded to an absolute improvement in depression due to active drug of 13.5% (95% CI, 12.4%-14.4%). The separation between treatment and placebo diminished near the tails of the distribution.

**Conclusions and Relevance:**

In this QTE analysis of pooled clinical trial data from the FDA, antidepressants were found to confer a small reduction in depression severity that was broadly distributed across participants with severe depression. Alternatively, if the assumptions behind the QTE analysis are not met, then the data are also compatible with antidepressants eliciting more complete response in a smaller subset of participants than is suggested by this QTE analysis.

## Key Points

**Question:**

What percentage of patients with severe depression experience improvement with antidepressant therapy and by how much?

**Findings:**

In this pooled secondary analysis of data from the US Food and Drug Administration that included 57 313 participants with severe depression from 232 randomized clinical trials of antidepressants for major depressive disorder, all quantiles of depression response were more favorable among drug-assigned participants, by 3% to 14%. These findings depend heavily on statistical assumptions.

**Meaning:**

These findings suggest that antidepressants may improve depression severity for a broad range of patients with severe depression, but for many patients, the magnitude of the reduction may be small.

## Teaser

This secondary analysis of pooled trial data evaluates the differences among randomized clinical trials in the outcomes of antidepressant monotherapy in patients with major depressive disorder.

## Introduction

Major depressive disorder (MDD) is a leading cause of global distress and disability.^1,2^ Antidepressants and psychotherapy are mainstays of MDD treatment.^3,4^ Most meta-analyses of antidepressant therapy randomized clinical trials focus on average treatment effect and/or its association with baseline characteristics.^5,6,7,8,9,10,11^ They generally find that patients with severe depression benefit from antidepressant therapy but only by a small amount on average, and this generates debate about whether use of antidepressants is worth the risk of adverse effects.^12,13,14^

This debate also depends on how antidepressant efficacy is distributed in populations and in individuals, which is less commonly studied. Both population-level distributions and individual-level distributions matter, and the conceptual differences between them are subtle but important as explained in the eAppendix in Supplement 1.

Stone et al^15^ recently estimated the distribution of antidepressant efficacy with a high-quality US Food and Drug Administration (FDA) data set and inferred that 15% of participants experience a robust response specific to active drug. That study used mixture models^16^ to estimate the distribution of antidepressant response. This study by Stone et al^15^ is an important contribution to the literature, but we do not share the statistical assumptions of that work. Specifically, their model assumes that the mixtures identify the 3 natural, distinct subtypes of MDD patient response, whereas our belief is that the identified mixtures may represent statistical artifact.^17^ We wrote to Stone et al^15^ for access to a shareable portion of their data to reestimate the population and individual antidepressant efficacy distributions using different assumptions.

We model the distribution of antidepressant response using a quantile treatment effect (QTE) framework.^18^ When estimating the distribution of individual antidepressant response, we assume rank similarity. Rank similarity is a popular premise in QTE analysis that allows extrapolation from the population-level distribution of response to the individual-level distribution of response.^19^ Under the rank similarity premise, the expected counterfactual placebo response at a given quantile of response among participants in the drug arm is modeled as the actual placebo response at that quantile. For example, it assumes that the 55th percentile of individuals who respond to drug therapy, had they instead been assigned to placebo, would have experienced the depression response of the 55th percentile of placebo-assigned participants and likewise for other quantiles.

Rank similarity is appropriate when the features that affect a participant’s rank within one treatment arm exert a similar effect in both arms. This is clinically plausible in the depression context because many of the same factors are highly clinically significant in determining the course of a patient’s depression in either arm of a trial. For instance, patients with worsening social circumstances tend to have worse depression responses compared with their same-arm peers in the trial whether assigned to drug or placebo. Similarly, patients whose depression before the trial had been long-standing tend to have worse depression responses compared with their same-arm peers whether assigned to drug or placebo. Rank similarity would be violated if factors that affect antidepressant responsiveness were more important for determining relative rank within an arm than features that affect the course of depression regardless of treatment assignment. A recent review stated that such factors have not been conclusively identified,^20^ although we note that this is an area in which developments in precision medicine are still emerging.^21,22,23^ If rank similarity is not met, then QTE analysis that assumes rank similarity will tend to underestimate the amount of heterogeneity in treatment effects.^24^ For interested readers unfamiliar with QTE analysis or who seek more intuition regarding the rank similarity premise in the present context, we include a miniature, fully explained example in the eAppendix, eTable 1, and eTable 2 in Supplement 1.

## Methods

### Data Acquisition

This quantile treatment effects study is a secondary analysis of pooled participant data. We obtained aggregate data through personal correspondence with the authors of Stone et al,^15^ who had analyzed individual participant data (IPD) of 73 388 participants of the 232 positive and negative randomized clinical trials of antidepressant monotherapy for MDD disorder submitted to the FDA between 1979 and 2016. Data analysis was conducted from August 16, 2022, to April 16, 2023. Because the underlying individual studies and certain aspects of the IPD are proprietary, there were significant limitations on what form of data Stone et al^15^ could share with us. Ultimately, we requested and received the following aggregate data for each combination of baseline depression severity and final depression severity: the number of individuals represented among their IPD in the treatment arm of any study and the number in the placebo arm of any study. These aggregate data imply a certain core set of IPD, such that we could extract for each participant their baseline depression severity, final depression severity, and whether they were assigned to the drug or placebo arms. In the aggregate data we received, depression severity had been converted to 17-item Hamilton Rating Scale for Depression (HAMD-17) equivalents as reported by Stone et al,^15^ rounded to the nearest integer. We did not receive any study-level information or any other data about participants. This was the minimum necessary information for completing our planned QTE analysis. Because we used aggregated, anonymized data, our study was determined exempt from institutional review board approval by Duke University Health System. The analysis plan was not preregistered and no analyses were prespecified. We followed the relevant portions of the Preferred Reporting Items for Systematic Reviews and Meta-analyses (PRISMA) reporting guideline.

### Statistical Analysis

#### Data Quality Testing and Processing

To test data completeness, we compared the counts of treatment-assigned and placebo-assigned participants in the aggregate data we received against the values reported by Stone et al.^15^ To test data consistency, we compared the range of baseline and final depression severity scores against the 0 to 52 range of the HAMD-17 scale. To test for risk of bias across studies introduced by the pooling process, we used a Wilcoxon rank sum test to compare baseline depression severity scores between treatment groups. When this test raised concern for a slight baseline imbalance, we used a literature-inspired filtering procedure of participants by baseline depression severity. A systematic review found that 20 is the most common HAMD-17 cutoff score for antidepressant trial inclusion among trials using the HAMD-17 scale; thus, we filtered out participants with baseline HAMD-17 scores less than 20.^25^ Then we again tested for baseline balance on the postfiltration set.

From the data obtained, we calculated 2 candidate measures of depression response since both percentage and absolute depression response are commonly used in studies.^26^ Percentage depression response was defined as 1 – F/B, expressed as a percentage, where F is the final depression severity and B is the baseline depression severity. Absolute depression response was defined as B – F. We used tests of rank similarity to guide which candidate measure of depression response to use in later parts of the study.

#### Testing Rank Similarity

We separately tested for rank similarity using our 2 candidate measures of depression response. We used the rank similarity test of Frandsen and Lefgren^19^ in which an available baseline attribute is tested for a significant interaction with the treatment arm when predicting the response, with the response separately tested as percentage depression response and absolute depression response. The available baseline attribute we used was baseline depression severity. Based on the results of these tests, we used percentage depression response as our chosen measure of depression response moving forward. A sensitivity analysis based on absolute depression response is presented in the eAppendix and eFigure in Supplement 1.

#### Quantile Treatment Effects

We calculated each quantile of percentage depression response separately in the treatment and placebo arms, at quantiles from the 5th percentile to 95th percentile, in increments of 5%. The QTEs were calculated as the difference between percentage depression response in the treatment vs placebo arms at a given quantile. The QTE 95% CIs were calculated by bootstrapping, using 10 000 iterations of the basic algorithm from the ci_quantile_diff function of the Hmisc R package, version 4.7-1. The QTEs were graphically plotted without covariates using the ci_qtet function of the qte R package, version 1.3.1 (R Foundation for Statistical Computing). Significance testing was performed via paired, 2-sided tests, with a significance threshold of *P* = .05.

## Results

### Data Quality

The aggregate data we received implied 71 393 participants, of whom 47 243 were assigned to drug and 24 150 to placebo. These values match the counts reported by Stone et al.^15^ All participants had an integer score for baseline depression severity and for final depression severity, and their range from 0 to 50 was within the 0 to 52 range of the HAMD-17 scale.

There was a slight and statistically significant difference in mean baseline depression severity between drug and placebo arms equivalent to 0.15 points on the HAMD-17 scale (*P* = 3.6 × 10^−5^ by Wilcoxon rank sum test); this difference in baselines could have arisen from variable randomization ratios among the individual studies composing our pooled data. To address this difference, we noted that participants with very low HAMD-17 scores may not meet the criteria for MDD and that many published randomized clinical trials of antidepressants only include participants with a HAMD-17 score of 20 or greater.^25^ Thus, we excluded 9115 drug-assigned participants and 4965 placebo-assigned participants with baseline HAMD-17 scores less than 20 from our pool, yielding a final analysis set of 57 313 participants. After this filtration, the difference in mean baseline depression severity between drug and placebo arms decreased to 0.037 HAMD-17 points and was no longer significant (*P* = .11 by Wilcoxon rank sum test). That is, the synthesis procedure used to produce our pooled data had some evidence of bias, which we were able to mitigate through filtering. Due to our study design and limitations in available data, some additional common tests were not applicable; the eAppendix in Supplement 1 provides details.

### Testing Rank Similarity

Since QTE analysis is most richly interpretable when rank similarity can be assumed, we tested whether our data and intended formulation were consistent with rank similarity. Following the method of Frandsen and Lefgren,^19^ we trained a linear model to predict percentage depression response from baseline depression severity, treatment arm, and the interaction between baseline depression severity and treatment arm. If there was a statistically significant interaction between baseline depression severity and treatment arm for prediction of percentage depression response in this model, then rank similarity would be rejected. When we applied this test, rank similarity was not rejected. Specifically, the interaction term between baseline depression severity and treatment arm in the model was not significant (*P* > .99). While this test cannot prove that rank similarity holds, the results we obtained from this test provide some statistical reassurance in the plausibility of the rank similarity assumption for our context.

Since there is not consensus in the literature about whether to define depression response as a percentage change from baseline as we have done vs an absolute change from baseline, we also tested for rank similarity using absolute change from baseline as the response variable. Under this alternative formulation, the interaction term between baseline depression severity and treatment arm in the model becomes significant (*P* = .003) (the eAppendix in Supplement 1 provides a discussion of evidence that the magnitude of this interaction is very small). At a minimum this finding indicates that a QTE analysis in which depression response is defined as an absolute change in baseline must adjust for baseline depression severity. We instead choose to use percentage depression response and not adjust for baseline depression severity.

### Quantile Treatment Effects

**Figure 1.**
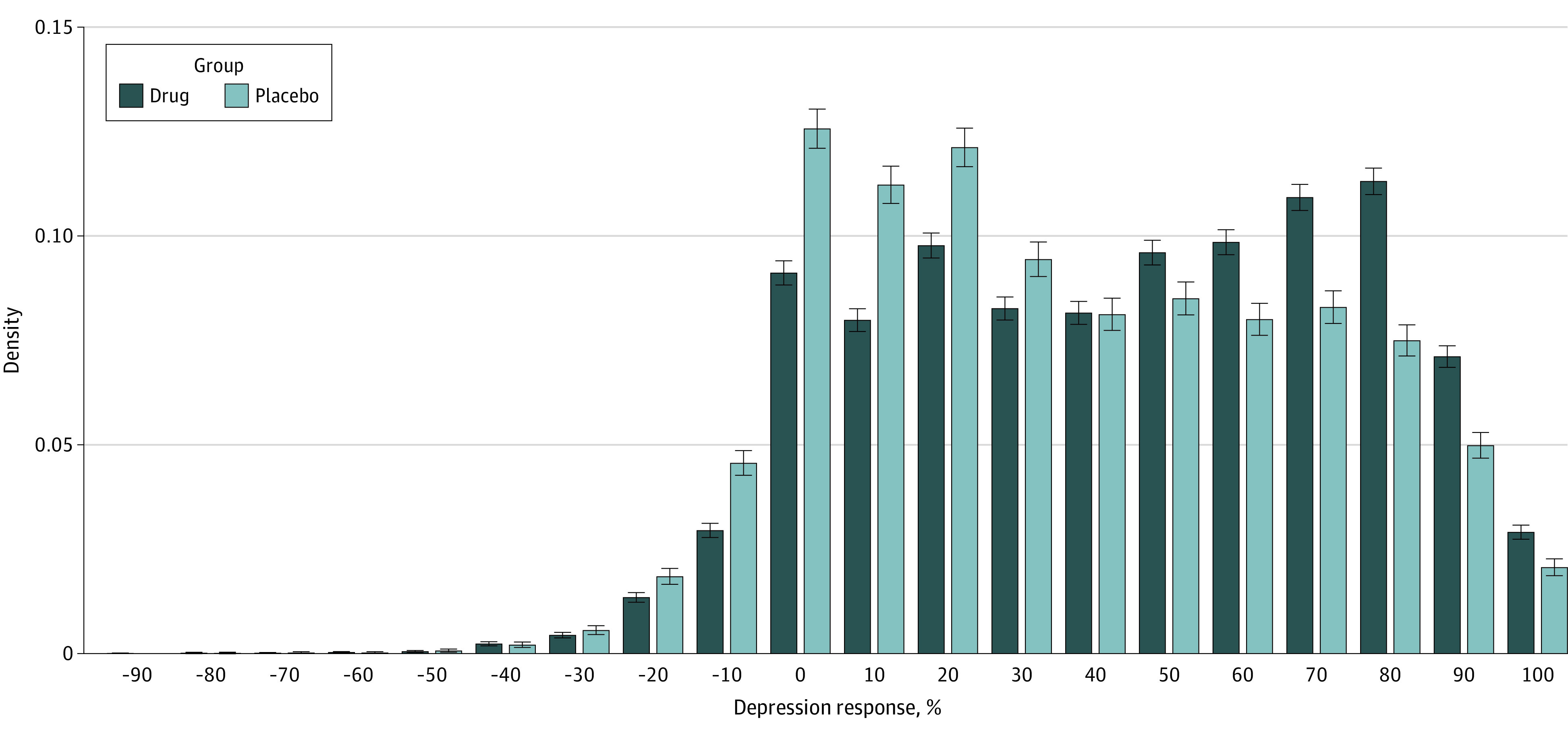
Distribution of Percentage Depression Response The distribution of percentage change of depression severity from baseline to trial end is depicted for each treatment arm. Error bars represent 95% CIs.

**Figure 2.**
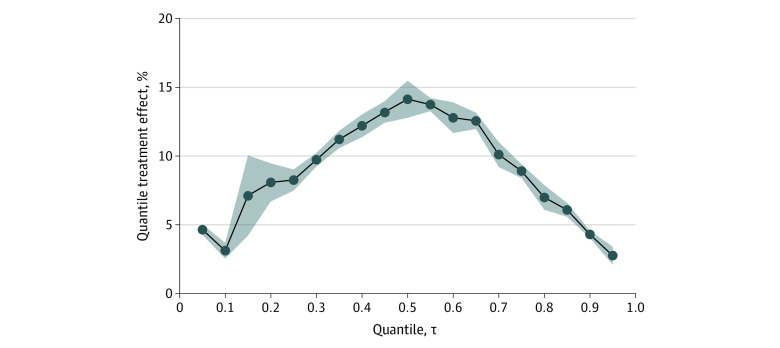
Quantile Treatment Effects of Antidepressants Compared With Placebo For each quantile (τ) of depression response, the difference between the treatment arm and placebo arm depression response at that quantile is shown, expressed as a percentage, along with its bootstrapped 95% CI.

Next, we characterized the estimated distribution of antidepressant response. We calculated the depression response distribution separately in treatment and control conditions (Figure 1), then calculated QTEs as the difference between these distributions (Figure 2). We observed that depression responses were more favorable in the treatment arm than in the placebo arm at all reported quantiles. At the 55th quantile, treatment arm participants had a final depression score that was 52.0% improved from baseline, and the corresponding value for placebo was 38.5% for a QTE of 13.5% (95% CI, 12.4%-14.4%), with values at other quantiles listed in the Table. The QTEs were greater in magnitude toward the center of the distribution and dissipated toward the tails. These results suggest that, if rank similarity holds, then participants at any quantile of depression response experienced at least some additional response from antidepressant treatment.

**Table.** QTEs at Selected Quantiles

Quantile within arm	Depression response, %		QTE (95% CI), %
	Treatment	Placebo	
0.95	91.3	88.5	2.8 (2.3-4.3)
0.90	85.2	80.8	4.4 (3.6-4.8)
0.85	80.0	73.9	6.1 (5.2-7.2)
0.80	75.0	68.0	7.0 (4.9-7.2)
0.75	70.8	61.9	8.9 (8.3-10.0)
0.70	66.7	56.0	10.7 (9.7-11.8)
0.65	61.9	50.0	11.9 (11.3-12.3)
0.60	57.1	44.0	13.1 (12.5-14.7)
0.55	52.0	38.5	13.5 (12.4-14.4)
0.50	46.7	33.3	13.3 (11.0-13.8)
0.45	40.9	28.0	12.9 (11.4-13.5)
0.40	35.0	22.7	12.2 (11.2-13.3)
0.35	29.6	18.5	11.1 (10.4-12.1)
0.30	23.8	14.3	9.5 (8.9-10.3)
0.25	18.2	10.0	8.2 (7.7-9.0)
0.20	13.0	5.0	8.0 (7.5-9.9)
0.15	7.1	0.0	7.1 (6.6-11.1)
0.10	0.0	−3.7	3.7 (3.6-7.4)
0.05	−5.0	−9.5	4.5 (4.0-5.1)

Abbreviation: QTE, quantile treatment effect.

## Discussion

We conducted a secondary analysis of pooled IPD from 232 randomized clinical trials submitted to the FDA for antidepressant monotherapy for MDD to characterize the distribution of antidepressant response by depression severity. We observed that the population distribution of depression responses was strictly superior in the treatment arm vs the placebo arm in this filtered data set of participants with severe baseline depression. All studied portions of the depression response distribution were at least as favorable in the treatment arm as in the placebo arm. The participants with the worst response receiving antidepressant treatment responded at least as favorably (in terms of percentage improvement in HAMD-17 scores) as the participants with the worst response receiving placebo. Likewise, the participants with the best response receiving antidepressant treatment responded at least as favorably as the participants with the best response receiving placebo, and similarly for everyone in between. The response was most pronounced in the center of the distribution. These observations do not depend on any special assumptions; they are a simple consequence of the raw quantile distributions of antidepressant treatment response.

We did not observe violations of rank similarity in our data when using the percentage definition of depression response. In contrast, when we defined depression response in terms of absolute improvement from baseline, there was a formal violation of rank similarity, although the magnitude of that observed violation was small. Our ability to test for rank similarity was limited. For instance, we only had the data to test for the potentially rank-distorting influence of a single potential moderator of treatment effect and not several other potential moderators that have been reported in the literature, such as brain network and perfusion patterns.^21,22,23^ If the rank similarity premise is indeed true, then our results are compatible with the possibility that all individuals with MDD may experience at least slightly better depression responses while receiving antidepressant therapy compared with placebo.

How should we interpret the fact that the middle of the quantile distribution is where the drug and placebo curves separate the most? Rank similarity permits the following interpretation: participants who would have had a typical, middling response to placebo (ie. a percentage change of HAMD-17 score from baseline to final response at or near the median of the distribution of placebo responses) stand to gain the most from active drug. In contrast, patients who would have had a robust response with placebo receive little additional benefit from active drug. Perhaps this is because those with a robust response to placebo need no further treatment. Similarly, nonresponders to placebo would have received little additional benefit from active drug. Perhaps this is because individuals who do not respond to placebo experience a particularly entrenched form of depression that also responds poorly to drugs.

These findings are exploratory and would need to be confirmed through specialized placebo run-in trials with a prolonged run-in duration (eg, the 6 weeks of placebo tested herein) followed by randomization of all run-in period participants, in contrast to common placebo run-in practices.^27^ The prediction is that partial responders in the run-in period would experience a greater benefit from active drug than would run-in nonresponders and run-in robust responders. If confirmed, future randomized clinical trials of antidepressants might increase their sensitivity by only randomizing partial responders of an adequate run-in period rather than the more common practice of randomizing nonresponders of a potentially inadequate run-in period.

Our analysis does not directly contradict that of Stone et al^15^ because that earlier work did not specifically preclude a large percentage of patients receiving small benefits from antidepressants and because we used a different response measure (percentage depression response) and a somewhat different data set (we excluded participants with low baseline HAMD-17 scores). Nonetheless, insofar as our study highlights the compatibility of our data with the possibility that all patients with severe depression may experience at least some improvement with antidepressant therapy, we provide a different perspective and emphasis than the focus of Stone et al^15^ on their estimated 15% of participants with substantial improvement while receiving antidepressants.

Analyses based on rank similarity tend to estimate response distributions as the broadest possible values that are consistent with the data. If rank similarity is only partly or approximately true, then the true distribution of antidepressant response would be more concentrated than the responses estimated herein. It would take a very large violation of rank similarity to arrive at responses as concentrated as those reported by Stone et al.^15^ While we believe that our statistical methods are well suited to uncovering distributions of response, it may be that the truth is somewhere between our estimates and those of Stone et al^15^ when it comes to individual patients.

### Strengths and Limitations

A strength of this study is its use of FDA data, which include both published and unpublished high-quality randomized clinical trials and their pooled IPD. Another strength is that the population-level findings do not depend on any special assumptions.

One limitation of the study is that individual-level findings depend on the rank similarity assumption, which is unproven. Other limitations include lack of associated data and therefore omitted analyses concerning adverse effects, long-term effects, demographic characteristic covariates, and study-specific information. The lack of study-specific information in particular means that we cannot account for between-study heterogeneity, which might otherwise affect the study-adjusted drug and placebo response curves and the magnitude of calculated QTEs. In keeping with other QTE analyses in the literature, we did not attempt an analysis of very finely resolved quantiles, such as the bottom 1% of the population.

Moreover, although we do not consider our choice to exclude patients with less severe depression from the analysis as a limitation to the internal validity of our study, this choice potentially limits the generalizability of our results to patients with mild depression. That exclusion further limits direct comparison of our results with those of Stone et al.^15^

## Conclusions

Among participants with baseline HAMD-17 scores of 20 or higher from 232 FDA trials for antidepressant monotherapy for MDD, all segments of the distribution of depression responses were more favorable with active treatment vs control. These findings suggest that if our statistical assumptions hold, then there is the possibility that nearly all patients with MDD with baseline HAMD-17 scores of 20 or greater experience at least some benefit from antidepressant therapy, although the magnitude of the response is more clinically meaningful in some patients than others. If our assumptions are not met, it is also possible that the same aggregate benefit is concentrated in substantially fewer patients. Regardless, estimating the percentage of patients who benefit from antidepressant therapy is a challenging task that depends on the statistical assumptions used.

## Notes

Supplement 1. **eAppendix.** Supplementary Information and QTE Example **eTable 1.** Simplified Illustration of QTEs Recovering Latent Natural Impact When Rank Assumptions Satisfied **eTable 2.** Simplified Illustration of QTEs Misidentifying Latent Natural Impact When Rank Assumptions Violated **eFigure.** Quantile Treatment Effects Framed as Absolute Change From Baseline

Supplement 2.
Data Sharing Statement

## References

[1] Rehm J, Shield KD. Global burden of disease and the impact of mental and addictive disorders. Curr Psychiatry Rep. 2019;21(2):10. doi:10.1007/s11920-019-0997-0 30729322

[2] Hasin DS, Sarvet AL, Meyers JL, . Epidemiology of adult *DSM-5* major depressive disorder and its specifiers in the United States. JAMA Psychiatry. 2018;75(4):336-346. doi:10.1001/jamapsychiatry.2017.4602 29450462 PMC5875313

[3] American Psychiatric Association. American Psychiatric Association Practice Guidelines for the Treatment of Psychiatric Disorders: Compendium. American Psychiatric Publishing; 2006.

[4] Guidi J, Fava GA. Sequential combination of pharmacotherapy and psychotherapy in major depressive disorder: a systematic review and meta-analysis. JAMA Psychiatry. 2021;78(3):261-269. doi:10.1001/jamapsychiatry.2020.3650 33237285 PMC7689568

[5] Fournier JC, DeRubeis RJ, Hollon SD, . Antidepressant drug effects and depression severity: a patient-level meta-analysis. JAMA. 2010;303(1):47-53. doi:10.1001/jama.2009.1943 20051569 PMC3712503

[6] Henssler J, Alexander D, Schwarzer G, Bschor T, Baethge C. Combining antidepressants vs antidepressant monotherapy for treatment of patients with acute depression: a systematic review and meta-analysis. JAMA Psychiatry. 2022;79(4):300-312. doi:10.1001/jamapsychiatry.2021.4313 35171215 PMC8851370

[7] Nunes EV, Levin FR. Treatment of depression in patients with alcohol or other drug dependence: a meta-analysis. JAMA. 2004;291(15):1887-1896. doi:10.1001/jama.291.15.1887 15100209

[8] Kirsch I, Deacon BJ, Huedo-Medina TB, Scoboria A, Moore TJ, Johnson BT. Initial severity and antidepressant benefits: a meta-analysis of data submitted to the Food and Drug Administration. PLoS Med. 2008;5(2):e45. doi:10.1371/journal.pmed.0050045 18303940 PMC2253608

[9] Rief W, Nestoriuc Y, Weiss S, Welzel E, Barsky AJ, Hofmann SG. Meta-analysis of the placebo response in antidepressant trials. J Affect Disord. 2009;118(1-3):1-8. doi:10.1016/j.jad.2009.01.029 19246102

[10] Posternak MA, Zimmerman M. Is there a delay in the antidepressant effect? a meta-analysis. J Clin Psychiatry. 2005;66(2):148-158. doi:10.4088/JCP.v66n0201 15704999

[11] Cipriani A, Furukawa TA, Salanti G, . Comparative efficacy and acceptability of 21 antidepressant drugs for the acute treatment of adults with major depressive disorder: a systematic review and network meta-analysis. Focus (Am Psychiatr Publ). 2018;16(4):420-429. doi:10.1176/appi.focus.16407 32021580 10.1176/appi.focus.16407 PMC6996085

[12] Moncrieff J, Kirsch I. Empirically derived criteria cast doubt on the clinical significance of antidepressant-placebo differences. Contemp Clin Trials. 2015;43:60-62. doi:10.1016/j.cct.2015.05.005 25979317

[13] Hengartner MP, Plöderl M. Estimates of the minimal important difference to evaluate the clinical significance of antidepressants in the acute treatment of moderate-to-severe depression. BMJ Evid Based Med. 2022;27(2):69-73. doi:10.1136/bmjebm-2020-111600 33593736

[14] Fountoulakis KN, Möller HJ. Are antidepressants clinically useful? conclusion of a decade of debate. World Psychiatry. 2014;13(2):201-202. doi:10.1002/wps.20112 24890076 PMC4102296

[15] Stone MB, Yaseen ZS, Miller BJ, Richardville K, Kalaria SN, Kirsch I. Response to acute monotherapy for major depressive disorder in randomized, placebo controlled trials submitted to the US Food and Drug Administration: individual participant data analysis. BMJ. 2022;378:e067606. doi:10.1136/bmj-2021-067606 35918097 PMC9344377

[16] McLachlan G, Lee S, Rathnayake S. Finite mixture models. Annu Rev Stat Appl. 2019;6:355-378. doi:10.1146/annurev-statistics-031017-100325

[17] Bauer DJ, Curran PJ. Distributional assumptions of growth mixture models: implications for overextraction of latent trajectory classes. Psychol Methods. 2003;8(3):338-363. doi:10.1037/1082-989X.8.3.338 14596495

[18] Heckman JJ, Smith J, Clements N. Making the most out of programme evaluations and social experiments: accounting for heterogeneity in programme impacts. Rev Econ Stud. 1997;64(4):487-535. doi:10.2307/2971729

[19] Frandsen BR, Lefgren LJ. Testing rank similarity. Rev Econ Stat. 2018;100(1):86-91. doi:10.1162/REST_a_00675

[20] García-Gutiérrez MS, Navarrete F, Sala F, Gasparyan A, Austrich-Olivares A, Manzanares J. Biomarkers in psychiatry: concept, definition, types and relevance to the clinical reality. Front Psychiatry. 2020;11:432. doi:10.3389/fpsyt.2020.00432 32499729 PMC7243207

[21] Rolle CE, Fonzo GA, Wu W, . Cortical connectivity moderators of antidepressant vs placebo treatment response in major depressive disorder: secondary analysis of a randomized clinical trial. JAMA Psychiatry. 2020;77(4):397-408. doi:10.1001/jamapsychiatry.2019.3867 31895437 PMC6990859

[22] Chin Fatt CR, Jha MK, Cooper CM, . Effect of intrinsic patterns of functional brain connectivity in moderating antidepressant treatment response in major depression. Am J Psychiatry. 2020;177(2):143-154. doi:10.1176/appi.ajp.2019.18070870 31537090

[23] Cooper CM, Chin Fatt CR, Jha M, . Cerebral blood perfusion predicts response to sertraline versus placebo for major depressive disorder in the EMBARC trial. EClinicalMedicine. 2019;10:32-41. doi:10.1016/j.eclinm.2019.04.007 31193824 PMC6543260

[24] Wüthrich K. A Comparison of two quantile models with endogeneity. J Bus Econ Stat. 2020;38(2):443-456. doi:10.1080/07350015.2018.1514307

[25] Zimmerman M, Clark HL, Multach MD, Walsh E, Rosenstein LK, Gazarian D. Symptom severity and the generalizability of antidepressant efficacy trials: changes during the past 20 years. J Clin Psychopharmacol. 2016;36(2):153-156. doi:10.1097/JCP.0000000000000466 26848791

[26] Leucht S, Fennema H, Engel R, Kaspers-Janssen M, Lepping P, Szegedi A. What does the HAMD mean? J Affect Disord. 2013;148(2-3):243-248. doi:10.1016/j.jad.2012.12.001 23357658

[27] Scott AJ, Sharpe L, Quinn V, Colagiuri B. Association of single-blind placebo run-in periods with the placebo response in randomized clinical trials of antidepressants: a systematic review and meta-analysis. JAMA Psychiatry. 2022;79(1):42-49. doi:10.1001/jamapsychiatry.2021.3204 34757405 PMC8581773

