# Supplementary material for: Use of Quantile Treatment Effects Analysis to Describe Antidepressant Response in Randomized Clinical Trials Submitted to the US Food and Drug Administration: A Secondary Analysis of Pooled Trial Data
Source: JAMA Netw Open. 2023 Jun 9;6(6):e2317714. doi: 10.1001/jamanetworkopen.2023.17714 (PMC10257092; doi:10.1001/jamanetworkopen.2023.17714)
Supplement: Supplement 1. — eAppendix. Supplementary Information and QTE Example eTable 1. Simplified Illustration of QTEs Recovering Latent Natural Impact When Rank Assumptions Satisfied eTable 2. Simplified Illustration of QTEs Misidentifying Latent Natural Impact When Rank Assumptions Violated eFigure. Quantile Treatment Effects Framed as Absolute Change From Baseline [file jamanetwopen-e2317714-s001.pdf]

Supplementary Online Content

Meyerson WU, Pieper CF, Hoyle RH. Use of quantile treatment effects analysis to describe antidepressant response in randomized clinical trials submitted to the US Food and Drug Administration: a secondary analysis of pooled trial data. *JAMA Netw Open*. 2023;6(6):e2317714. doi:10.1001/jamanetworkopen.2023.17714

eAppendix. Supplementary Information and QTE Example

eTable 1. Simplified Illustration of QTEs Recovering Latent Natural Impact When Rank Assumptions Satisfied

eTable 2. Simplified Illustration of QTEs Misidentifying Latent Natural Impact When Rank Assumptions Violated

eFigure. Quantile Treatment Effects Framed as Absolute Change From Baseline

This supplementary material has been provided by the authors to give readers additional information about their work.

© 2023 Meyerson WU et al. *JAMA Network Open.*

eAppendix. Distribution of Effects and QTE Example

Population vs individual distributions of antidepressant response

The distribution of population-level effects of antidepressants, as considered here, answers questions like the following: how does the depression response of the worst-responding drug-assigned participants compare to the depression response of the worst-responding participants on placebo? How does the depression response of the typically-responding and best-responding participants compare between arms? At the population level, healthcare systems might increase their investment in antidepressant access, for instance, if it were discovered that antidepressant efficacy is distributed within the population in a way that could be expected to reduce the total number of hospitalizations.

The distribution of individual-level effects of antidepressants answers questions like the following: If an individual is assigned to drug, how likely is it that their depression would improve or worsen and by how much compared to if they had instead been assigned to placebo? Suppose that the population-level effects of antidepressants were such that the worst-responding, typically-responding, and best-responding participants on drug all responded more favorably than the correspondingly-ranked participants on placebo. In this case it could still be theoretically possible that the worst-responding participant on drug would have been the best-responding participant if he or she had instead been assigned to placebo and therefore suffered a large individual harm from antidepressants.

The population-level distribution of antidepressant efficacy is straightforward to calculate from pooled participant data of randomized clinical trials, by comparing the cumulative density function of depression response in drug vs placebo, although few studies do. In contrast, the distribution of individual-level antidepressant effects can only be estimated with additional assumptions because a single individual is only assigned to either one of drug or placebo (except in cross-over trials, which pose their own difficulties in antidepressant studies).

Illustrated QTE example

To illustrate QTE analysis in depression, consider a highly simplified example (eTable 1 and eTable 2). Suppose that, unbeknownst to investigators, there are three kinds of natural histories of participants enrolled in antidepressant trials who experience different depression response trajectories for reasons that are external to the trial. For concreteness in this illustration let us say that these three natural histories correspond to those who experience improving, stable, or worsening social circumstances over the course of the trial for reasons external to the trial. Further suppose that one participant of each of these three types is randomized to drug and one of each type is randomized to placebo, such that we have I_d, S_d, and W_d and I_p, S_p, and W_p as participants with **I**mproving, **S**table, and **W**orsening social circumstances in **d**rug and **p**lacebo arms, respectively**.** For instance, perhaps I_d and I_p had become depressed before the trial due to being laid off from work, but then during the course of the trial, their employers' financial circumstances improved and I_d and I_p were rehired. Meanwhile, perhaps W_d and W_p experienced the sudden death of a beloved family member during the course of trial and S_d and S_p

© 2023 Meyerson WU et al. *JAMA Network Open.*

experienced no specific social changes. Let us say that I_d and I_p together makes a natural pair since we are assuming in this illustration that they would have the same natural histories, and likewise for S_d with S_p and for W_d with W_p.

A particularly appropriate method here to uncover the causal impact of the antidepressant on an individual drug-assigned participant is to compare his or her depression response with that of the placebo-assigned participant with the same expected natural history. However, since the investigators are not systematically recording the changing social circumstances of the participants (and more generally, since expected natural history is not directly observable), they cannot directly read off which participant in the placebo arm has the same expected natural history as a given participant of the treatment arm. Assumptions of preserved rank allows the investigators to nonetheless identify pairings between drug and placebo participants that are assumed to be the latent natural pairings. If it can be assumed that the rehired participant of a given arm experiences a better depression response than the socially stable participant of that arm, who in turn experiences a better depression response than the participant of that arm who experienced the death of a family member, then the way to recover the natural pairings in the data is to match the best-responding drug-assigned participant with the best-responding placebo-assigned participant, the second-best-responding drug-assigned participant with the second-best-responding placebo-assigned participant, and so on (eTable 1). This is an assumption, and it is also possible that the interaction between natural histories and drug-specific-response could upset the rankings of the expected natural histories in the treatment group compared to those of the placebo group (eTable 2). The researchers in this simplified example would be more justified in using QTE analysis if they successfully argued that from clinical experience, they would not expect a newly-grieving participant to fare better than a newly-rehired participant from the same arm of an antidepressant clinical trial. Real life is more complicated that this illustrative example but the same principles apply.

© 2023 Meyerson WU et al. *JAMA Network Open.*

eTable 1: Simplified illustration of QTEs recovering latent natural impact when rank assumptions satisfied

**Depression response rank within arm**	**Drug participant**	**Drug response**	**Placebo participant**	**Placebo response**	**QTE pairing**	**Latent natural pairing**	**QTE estimated impact of drug**	**Latent natural impact of drug**
1 of 3	I_d	50%	I_p	45%	(I_d, I_p)	(I_d, I_p)	5%	5%
2 of 3	S_d	30%	S_p	15%	(S_d, S_p)	(S_d, S_p)	15%	15%
3 of 3	W_d	0%	W_p	-10%	(W_d, W_p)	(W_d, W_p)	10%	10%

I_d, ***S_d***, and ***W_d*** and ***I_p***, ***S_p***, and ***W_p*** as participants with **I**mproving, **S**table, and **W**orsening social circumstances in **d**rug and **p**lacebo arms, respectively. Here, the most-improved (rank 1 of 3) participant in the drug arm is I_d whose depression improves by 50% on drug, and the most-improved participant in the placebo arm is I_p whose depression improves by 45% on placebo. QTE analysis pairs participants I_d and I_p with each other since they have the same ranks within their respective arms and thereby estimates that drug did improve I_d by an additional 5% and would have improved I_p by an additional 5% if he or she had instead been assigned to drug. This pairing also recovers the latent natural pairing of the two individuals who, unbeknownst to investigators, are experiencing improving social circumstances.

© 2023 Meyerson WU et al. *JAMA Network Open.*

eTable 2: Simplified illustration of QTEs misidentifying latent natural impact when rank assumptions violated

Depression response rank within arm	Drug participant	Drug response	Placebo participant	Placebo response	QTE pairing	Latent natural pairing	QTE estimated impact of drug	Latent natural impact of drug
1 of 3	W_d	50%	I_p	45%	(W_d, I_p)	(W_d, W_p)	5%	60%
2 of 3	S_d	30%	S_p	15%	(S_d, S_p)	(S_d, S_p)	15%	15%
3 of 3	I_d	0%	W_p	-10%	(I_d, W_p)	(I_d, I_p)	10%	-45%

I_d, S_d, and W_d and I_p, S_p, and W_p as participants with **I**mproving, **S**table, and **W**orsening social circumstances in **d**rug and **p**lacebo arms, respectively. Here, the most-improved (rank 1 of 3) participant in the drug arm is W_d whose depression improves by 50% on drug, and the most-improved participant in the placebo arm is I_p whose depression improves by 45% on placebo. QTE analysis pairs participants W_d and I_p with each other since they have the same ranks within their respective arms and thereby estimates that drug did improve W_d by an additional 5% and would have improved I_p by an additional 5% if he or she had instead been assigned to drug. In this case, QTE analysis does not recover the pairings between individuals from the same latent natural classes. If we instead compare individuals across arms from the same latent natural categories, then we see that W_d was improved by 60% relative to the placebo individual with similarly worsening social circumstances, and that if I_p had received drug, he or she might have had a 45% worse depression response corresponding to that of the drug-assigned individual with similarly improving social circumstances. However, note that the setup of eTable 2 (unlike that of eTable 1) requires the drug-assigned participant with worsening social circumstances to have a better depression response than the drug-assigned patient with improving social circumstances, but this is contrary to clinical expectations.

Standard analyses not included due to study design and limitations in data

Due to our study design as a re-analysis of previously pooled data, we present no results related to study selection. Due to the proprietary nature of the underlying data in our study, the aggregate data we received did not contain study-level information. Therefore, we present no results related to study-level information, such as study characteristics, risk of bias within studies, results of individual studies, availability of IDP per study, or heterogeneity between studies. Interested readers can refer to Stone *et al.* for their results related to these aspects of the underlying data.

Magnitude of observed departure from rank similarity in absolute reduction case

In the main manuscript, we noted that in the linear model that predicts absolute depression response from baseline depression score, treatment arm, and their interaction, the interaction term was statistically significant, providing nominal evidence of departure from rank similarity when defining depression response as absolute (but not percentage) improvement. Here we test the magnitude of this response in two ways. First, we examined the magnitude of the interaction coefficient from the linear model. The interaction coefficient was found to be 0.08, meaning each additional point of baseline depression severity on the HAMD-17 scale predicts an additional 0.08 HAMD-17 points of response in drug vs placebo. Second, a potentially more robust test to gain insight into the importance of the interaction term is through comparison of

© 2023 Meyerson WU et al. *JAMA Network Open.*

the R-squared of nested models with and without the interaction term. The R-squared for a linear model that models the absolute change in depression severity from the baseline depression score and treatment arm is 0.0414. When the interaction term is added to the model, the model's R-squared increases by a paltry 0.0002, to 0.0416. This latter observation suggests that the observed departures from rank similarity using the absolute definition and the baseline depression scores are more so nominal rather than meaningful.

Distribution of antidepressant responses when using absolute depression response as definition of depression response

In the main manuscript, QTEs are calculated using percentage depression response as the definition of depression response. As a further sensitivity test, we re-calculated QTEs using absolute improvement definition of depression response. The results of this sensitivity analysis are qualitatively similar to those obtained through the percentage improvement definition of depression response, with favorable QTEs in the direction of drug at all quantiles which peak in the middle quantiles (eFigure). These results indicate that, at the population-level among participants with severe depression, the study population assigned to drug has a strictly superior distribution of depression response compared to the placebo-assigned population regardless of whether depression response is defined in terms of absolute vs percentage improvement from baseline. Here however we would be less justified in interpreting QTEs as individual-level effects of drug or even effects of drug on a subpopulation at a given quantile because of the (albeit small) violations in rank similarity we previously observed in rank similarity testing using the absolute improvement definition of depression response.

© 2023 Meyerson WU et al. *JAMA Network Open.*

eFigure: Quantile treatment effects framed as absolute change from baseline

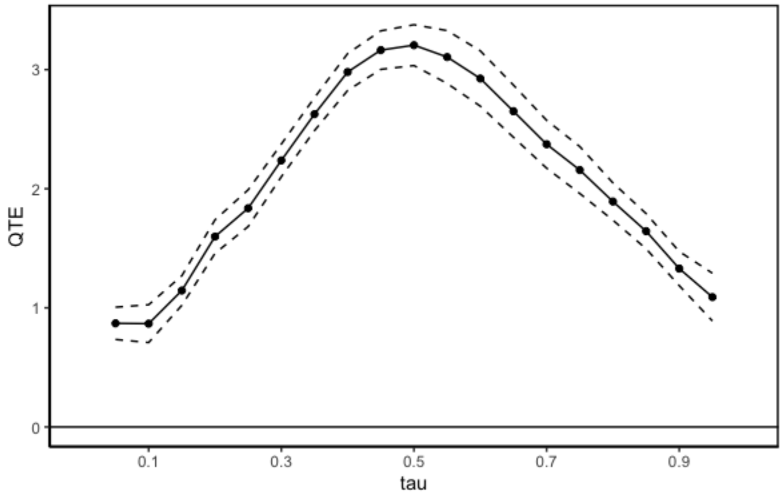

© 2023 Meyerson WU et al. *JAMA Network Open.*
