# Supplementary material for: Use of Quantile Treatment Effects Analysis to Describe Antidepressant Response in Randomized Clinical Trials Submitted to the US Food and Drug Administration: A Secondary Analysis of Pooled Trial Data
Source: JAMA Netw Open. 2023 Jun 9;6(6):e2317714. doi: 10.1001/jamanetworkopen.2023.17714 (PMC10257092; doi:10.1001/jamanetworkopen.2023.17714)
Supplement: Supplement 2. — Data Sharing Statement [file jamanetwopen-e2317714-s002.pdf]

Data Sharing Statement

Meyerson. Use of Quantile Treatment Effects Analysis to Describe Antidepressant Response in Randomized Clinical Trials Submitted to the US Food and Drug Administration. *JAMA Netw Open*. Published June 09, 2023. doi:10.1001/jamanetworkopen.2023.17714

Data
Data available: No

Additional Information
Explanation for why data not available: No new data was generated for this metanalysis.
